# Supplementary material for: Altered developmental programs and oriented cell divisions lead to bulky bones during salamander limb regeneration
Source: Nat Commun. 2022 Nov 14;13:6949. doi: 10.1038/s41467-022-34266-w (PMC9663504; doi:10.1038/s41467-022-34266-w)
Supplement: Supplementary file 1 — Supplementary Information [file 41467_2022_34266_MOESM1_ESM.pdf]

SUPPLEMENTAL NOTE

Formation and ossification of cartilage share structural, cellular and molecular characteristics with mammals during salamander limb development

To compare skeletogenesis during regeneration and development, we characterised the formation of skeletal elements during salamander limb development in detail. For this purpose, we disclosed stereotypical morphogenesis of forelimb cartilaginous elements in larval and adult limbs from the newt *Pleurodeles waltl* by reconstructing cartilage and bone structures employing 3D-rendered micro-CT scans (Fig. 1, 2, S1). Osteogenesis was observed in the humerus starting at larval stage 55a (Fig. 2A,B, S1A). Interestingly, the bones ossified along the wall of cartilage elements without resorption of the latter (Fig. 2B; Fig. S3B (control limb)). We confirmed micro-CT data using immunohistochemical analyses with antibodies against SOX9, Collagen type II (COL2A1) (Fig. S2), whereas cartilaginous and mineralised areas were visualised with Alcian Blue/Von Kossa staining (Fig. 1E,G, Fig. S3B). Thus, intramembranous bone formation (bone formed without cartilage intermediate) seems to be uncoupled from the endochondral bone formation (bone formed upon cartilage erosion) during larval limb growth.

Closer to metamorphosis (stage 56), abrupt ossification occurs progressively, and eventually leads to fully ossified bones in adult *Pleurodeles* (Fig. 1A, see control limb). Overall, these results show that, in contrast to mammals, intramembranous and endochondral ossifications of the skeletal elements in the salamander forelimb are substantially uncoupled during larval stages. At the same time, the major steps of developmental chondrogenesis resemble those in mammals, including appearing Sox9- and Col2-positive chondrocytes following their hypertrophy in a proximo-distal sequence (Fig. S2).

Next, we tested whether the molecular cues of the PTHrP/Ihh loop, known to drive the elongation of bones during mammalian development, are reiterated during salamander development. We first identified *Pth*, *PTHrP*, *Gli1*, *Shh* and *Ihh* in the *Pleurodeles* genome and transcriptome [1] (Fig. S3A). We subsequently analyzed their RNA expression patterns in *Pleurodeles* forelimbs at stage 55a. We could neither detect *Pth* nor *Shh* in the elongating forelimbs, but RNAscope revealed that salamander skeletal elements expressed *PTHrP*, *Gli1* and *Ihh* domains in an analogous pattern to mammals (Fig. S3C, S8). However, we noticed that perichondrium appeared as another source of PTHrP (Fig. S3C, S8), thus likely complementing the relatively low expression of PTHrP by round chondrocytes. This might, in theory, explain a rather unorganised and reduced zone of flat chondrocytes in larval limbs (Fig. S2).

In line with this severely reduced zone of flat chondrocytes known to be highly proliferative in mice, extensive EdU labelling revealed no specific domain of highly-proliferative chondrocytes (Fig. S5C-D). At the same time, most of the EdU doublets were oriented transversally, following the logic of oriented clonal patterns observed during cartilage formation in mice before the formation of the stem cell niche and the growth plate. We obtained similar results in the developing limbs of the axolotl, *Ambystoma mexicanum* (Fig S5A).

1 Elewa, A. *et al.* Reading and editing the Pleurodeles waltl genome reveals novel features of tetrapod regeneration. *Nat Commun* **8**, 2286, doi:10.1038/s41467-017-01964-9 (2017).

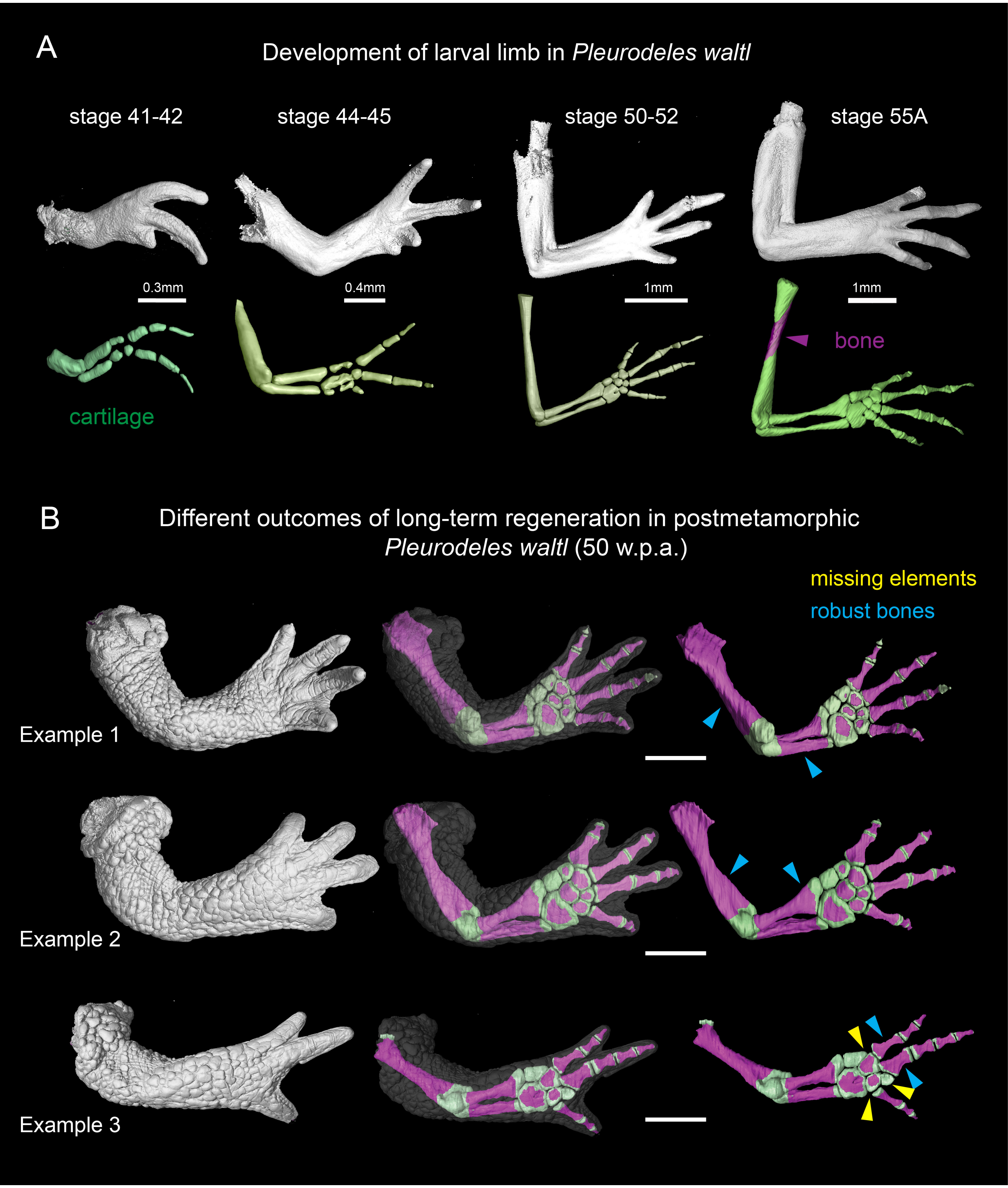

A
Development of larval limb in *Pleurodeles waltl*
stage 41-42
stage 44-45
stage 50-52
stage 55A
0.3mm
0.4mm
1mm
1mm
bone
cartilage
B
Different outcomes of long-term regeneration in postmetamorphic *Pleurodeles waltl* (50 w.p.a.)
missing elements
robust bones
Example 1
Example 2
Example 3

Figure S1. Micro-CT reconstruction of developing and regenerating limbs in *Pleurodeles waltl*

(A) Micro-CT scans and 3D models of physiologically growing forelimbs in larval *Pleurodeles waltl* analyzed at various stages. Magenta arrow points at the forming bone in the stage 55a, before the outset of metamorphosis. Green color represents cartilage. Scale bars larval limbs, indicated in figure (from left to right: 300 µm, 400 µm, 1 mm, 1 mm).

(B) Micro- CT scans and segmented 3D models of different outcomes of long-term regeneration in post-metamorphic *Pleurodeles waltl*. Yellow arrows point at missing/fused skeletal elements. Blue arrows point at remarkably robust regenerated bones. Green color represents the cartilage and magenta color represents bone. Scale bars, 4mm.

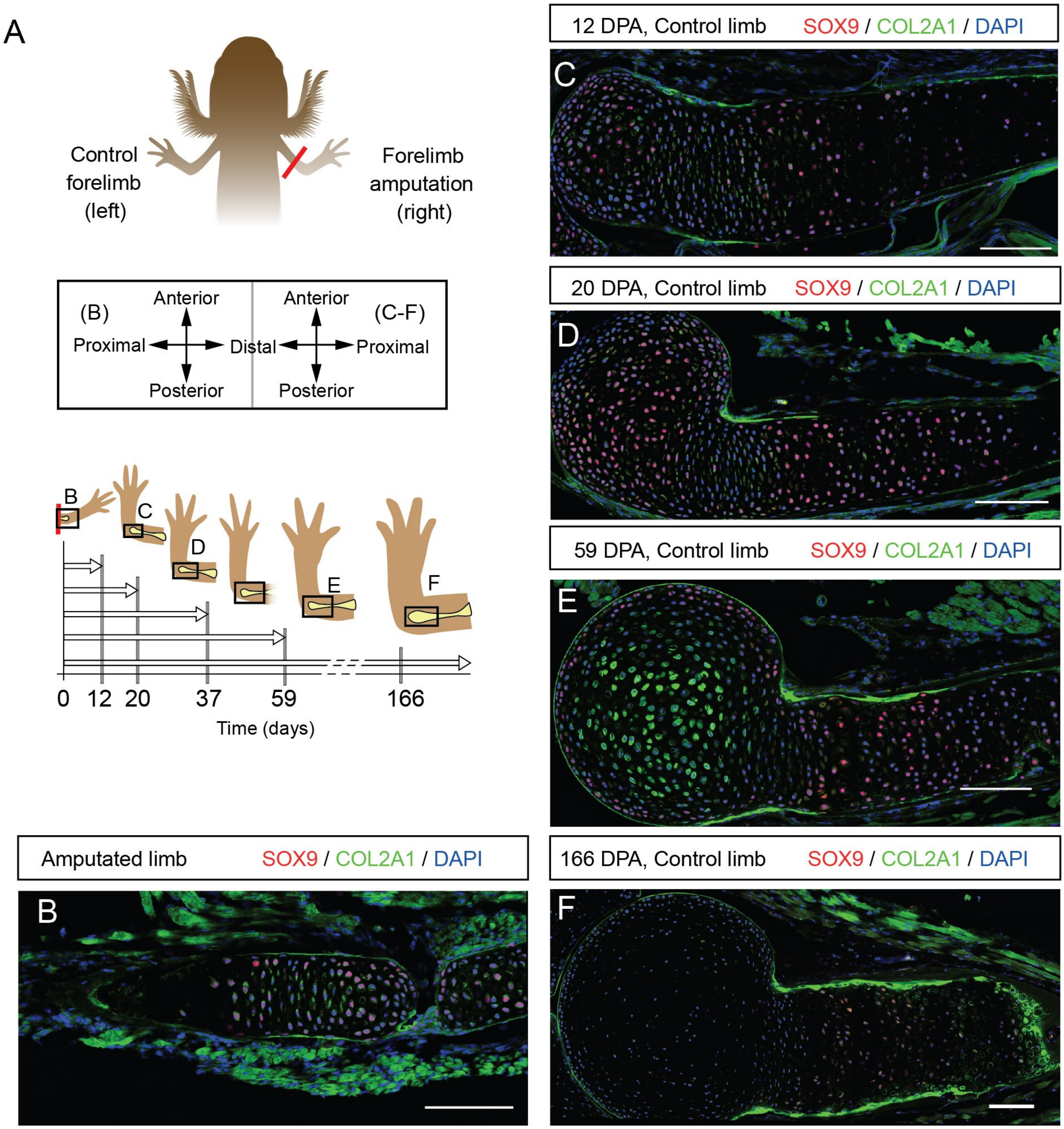
A
Control forelimb (left)
Forelimb amputation (right)
(B)
Anterior
Proximal
Distal
Posterior
(C-F)
Anterior
Proximal
Posterior
B
C
D
E
F
0
12
20
37
59
166
Time (days)
12 DPA, Control limb SOX9 / COL2A1 / DAPI
C
20 DPA, Control limb SOX9 / COL2A1 / DAPI
D
59 DPA, Control limb SOX9 / COL2A1 / DAPI
E
Amputated limb SOX9 / COL2A1 / DAPI
B
166 DPA, Control limb SOX9 / COL2A1 / DAPI
F

Figure S2. Histological analysis of cartilage growth and ossification validates micro-CT analysis

(A) Experimental outline of regeneration experiment in *Pleurodeles waltl* larvae showing the contralateral unamputated control limbs (regeneration part is summarised in Figure 6). Larvae at stage 54 received unilateral amputations. The regenerating limbs (see Fig. 6) and contralateral controls (present Figure) were collected for analysis at the selected time points. The limbs used as controls and the region shown in B-F are marked above the timeline.

(B) The amputated limb was used as an uninjured, control limb, showing that at the stage 54 larvae, the humerus is characterised by $SOX9^{+}/COL2A1^{+}$ emerging chondrocytes. Scale bar, 200µm.

(C) At 12 days post-amputation (d.p.a.), the contralateral control humerus contains a majority of $SOX9^{+}/COL2A1^{+}$ chondrocytes. Scale bar, 200µm.

(D) At 20 days post-amputation (d.p.a.), the contralateral control humerus maintains a majority of $SOX9^{+}/COL2A1^{+}$ chondrocytes. Scale bar, 200µm.

(E) At 59 days post-amputation (d.p.a.), the $SOX9^{+}$ expression in the contralateral control humerus decreased while a strong $COL2A1^{+}$ signal was detected in the central part of the diaphysis. Scale bar, 200µm.

(F) At 166 days post-amputation (d.p.a.), we could not detect $SOX9^{+}$ nuclei in the contralateral control humerus, and the $COL2A1^{+}$ signal was detected in the hypertrophic chondrocytes. Scale bar, 200µm.

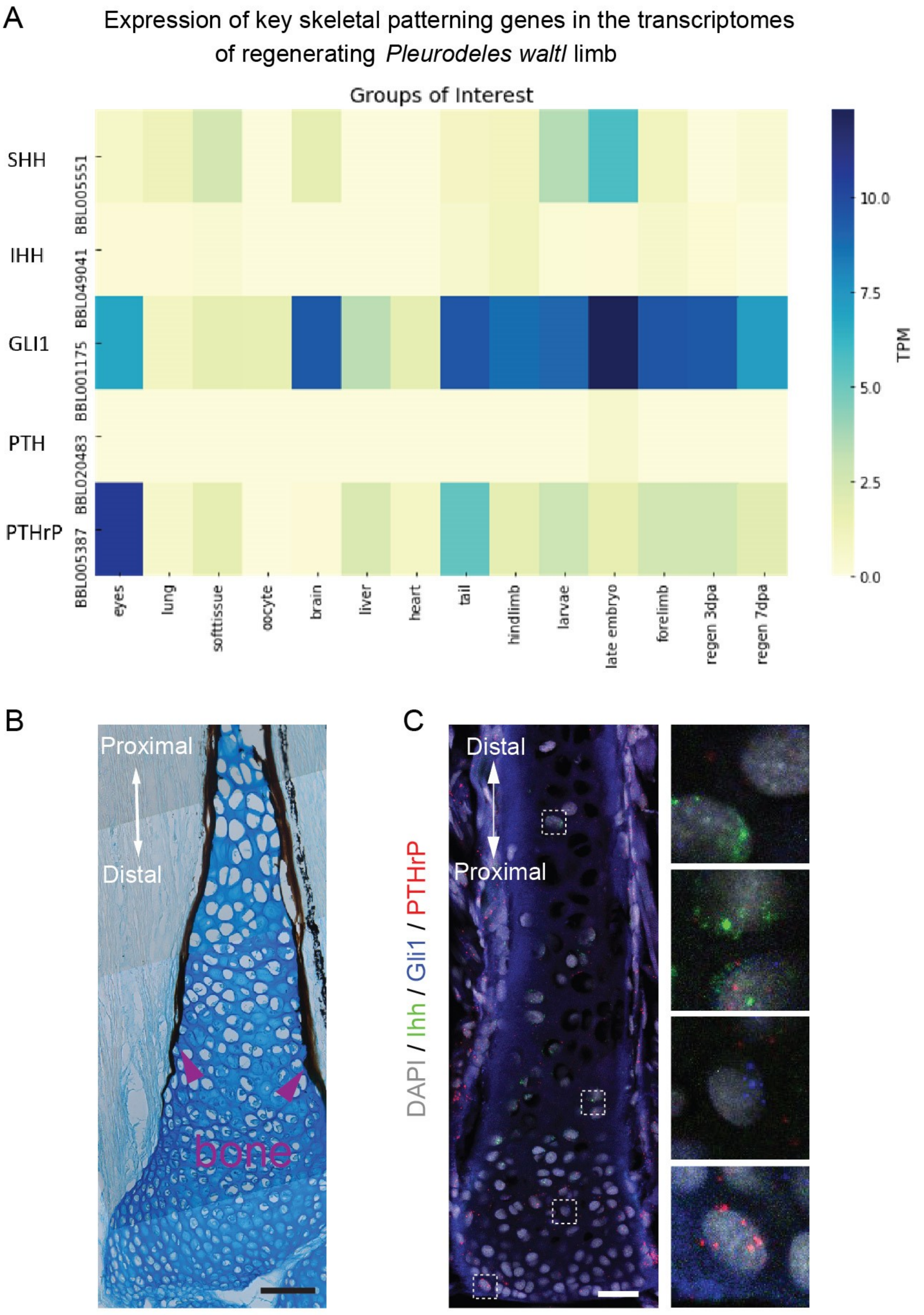
A
Expression of key skeletal patterning genes in the transcriptomes of regenerating *Pleurodeles waltl* limb
Groups of Interest
SHH
BBL005551
IHH
BBL049041
GLI1
BBL001175
PTH
BBL020483
PTHrP
BBL005387
eyes
lung
softtissue
oocyte
brain
liver
heart
tail
hindlimb
larvae
late embryo
forelimb
regen 3dpa
regen 7dpa
TPM
10.0
7.5
5.0
2.5
0.0
B
Proximal
Distal
bone
C
Distal
Proximal
DAPI / Ihh / Gli1 / PTHrP

Figure S3. The formation and ossification of cartilage in salamander limb development share structural, cellular and molecular characteristics with mammals

(A) Tissue-specific RNAseq quantification of genes traditionally linked to the cartilage development: *Shh, Ihh, Gli1, Pth and PTHrP*. Transcripts per million (TPM).

(B) Alcian blue / Von Kossa staining revealing cartilage and bone in a physiologically growing radius of a *Pleurodeles waltl* larva (stage 55a). Scale bar, 100µm.

(C) Mapping the expression of *Ihh, Gli1,* and *PTHrP* in the developing (amputated limb corresponding to Fig. S2B) forelimb cartilage of *Pleurodeles waltl*.

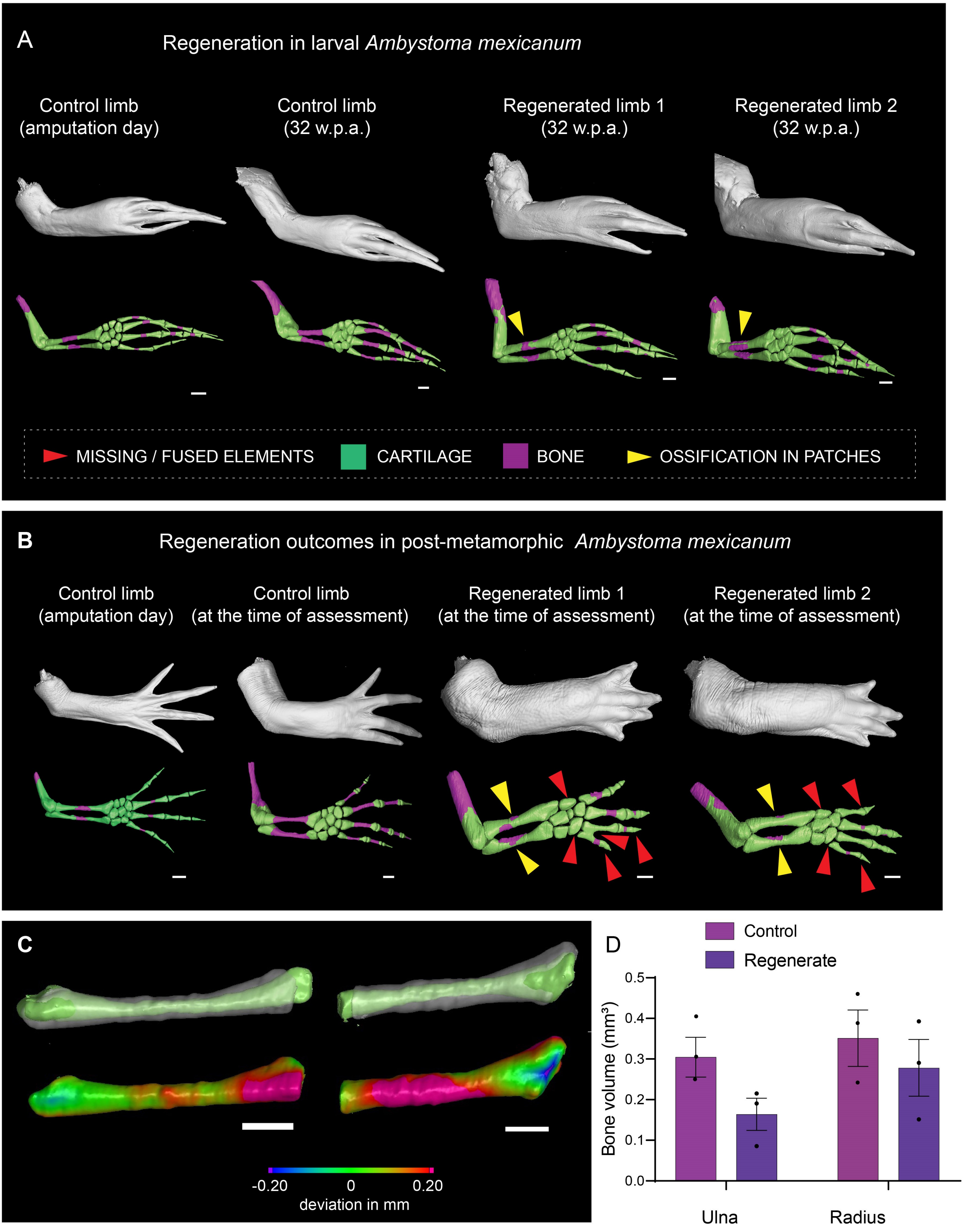
A
Regeneration in larval Ambystoma mexicanum
Control limb (amputation day)
Control limb (32 w.p.a.)
Regenerated limb 1 (32 w.p.a.)
Regenerated limb 2 (32 w.p.a.)
MISSING / FUSED ELEMENTS
CARTILAGE
BONE
OSSIFICATION IN PATCHES
B
Regeneration outcomes in post-metamorphic Ambystoma mexicanum
Control limb (amputation day)
Control limb (at the time of assessment)
Regenerated limb 1 (at the time of assessment)
Regenerated limb 2 (at the time of assessment)
C
-0.20
0
0.20
deviation in mm
D
Control
Regenerate
Bone volume (mm³)
0.0
0.1
0.2
0.3
0.4
0.5
Ulna
Radius

Figure S4. Skeletal elements in regenerating *Ambystoma mexicanum* limb differ from analogous skeletal parts forming during physiological growth

(A) Micro-CT scans and segmented 3D models of larval limb regeneration in *Ambystoma mexicanum*. On the left, the control limbs at the time of amputation and collection (32 w.p.a.) are shown. On the right, the outcomes of long-term limb regeneration are shown. Regenerated skeletal elements appear robust compared to controls, and ossification outset develops within the cortical bone in patches. Yellow arrows point to ossification patches. Scale bars, 1mm.

(B) Micro-CT scans and segmented 3D models of post-metamorphic limb regeneration in *Ambystoma mexicanum*. On the left, the control limbs at the times of amputation and collection are shown. On the right, various outcomes of long-term limb regeneration are shown. Note the robustness of the regenerated skeletal elements and ossification outset occurring in patches within the cortical and sub-cortical bone. Red arrows point at missing/fused or mis-patterned skeletal elements. Yellow arrows point to ossification patches. Scale bars, 1mm.

(C) 3D comparisons of shapes of normally developed and regenerated skeletal elements from the forelimb of larval *Ambystoma mexicanum*. Note that regenerated skeletal parts possess larger diameter, the shape differences between control and regenerated skeletal elements are visualised as a heatmap of shape deviation. Scale bars, 1mm.

(D) Quantification of the volumes occupied by ossified bone in the radius and ulna of fully regenerated (32 w.p.a.) versus corresponding control limbs of larval *Ambystoma mexicanum*. Regenerated limbs contained a similar volume of bone than uninjured controls at this stage, although ossification may still be ongoing (2-way ANOVA: n.s. Interaction, p=0.5780; n.s. Zeugopodial element, p=0.2053; n.s. Regeneration, p=0.1047). n=3 limbs per condition. Data are presented as mean values +/- SEM.

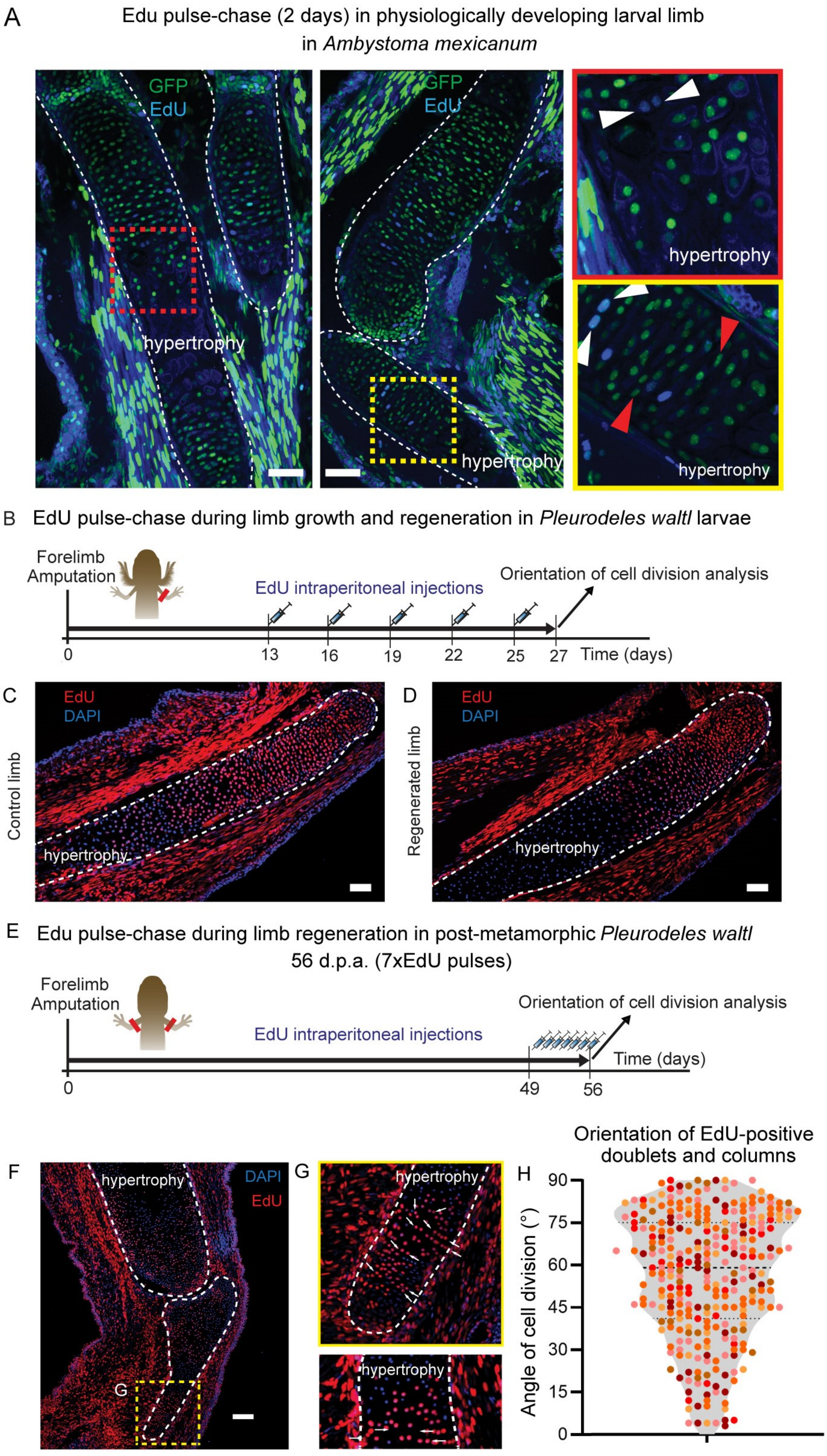
A
Edu pulse-chase (2 days) in physiologically developing larval limb in *Ambystoma mexicanum*
GFP
EdU
hypertrophy
B EdU pulse-chase during limb growth and regeneration in *Pleurodeles waltl* larvae
Forelimb Amputation
EdU intraperitoneal injections
Orientation of cell division analysis
0
13
16
19
22
25
27
Time (days)
C
D
EdU
DAPI
Control limb
Regenerated limb
E Edu pulse-chase during limb regeneration in post-metamorphic *Pleurodeles waltl*
56 d.p.a. (7xEdU pulses)
49
56
F
G
H
Orientation of EdU-positive doublets and columns
Angle of cell division (°)
90
75
60
45
30
15
0

Figure S5. Transversal chondrocytic cell divisions occur during development and regeneration of rod-shaped cartilages in salamanders

(A) Analysis of cell divisions after an EdU pulse (2 days post-injection) in regenerating larval limb of *Ambystoma mexicanum*. Red and yellow dotted lines mark the areas that are magnified on the right side. Red arrows point at typically flattened cells in proximity to the hypertrophic area. White arrows point at divided EdU-positive cells. Note the absence of highly proliferating clones as is typical for the mammalian long bones. White dotted lines outline the cartilage elements. Scale bar, 100µm.

(B) Experimental design of EdU pulse-chase analysis in both the physiologically growing and regenerating larval limb of *Pleurodeles waltl* at stage 52a.

(C) EdU-pulsed control limbs show no abundance of cell divisions in the area close to the ossification (hypertrophy). White dotted lines outline the cartilage elements. Scale bar, 100µm.

(D) EdU-pulsed regenerated limbs show no abundance of cell divisions in the area close to the ossification (hypertrophy). Note the decreased signal of EdU due to more or faster divisions in the regenerated limb compared to the control (C). White dotted lines outline the cartilage elements. Scale bar, 100µm.

(E) Experimental design of EdU pulse-chase analysis in regenerating post-metamorphic limb of *Pleurodeles* (stage 56, juvenile) at 8 weeks after amputation.

(F) The area close to ossification (hypertrophy of chondrocytes) does not show many cell divisions. Yellow dotted square marks the area magnified in (G) that is further magnified below. White arrows point at EdU-positive doublets that have a transverse orientation. White dotted lines outline the cartilage elements. Scale bar, 200µm.

(H) Violin plot showing the quantification of cell division orientations in EdU-treated limbs. Note that transverse orientation is predominant. Each data point represents the orientation of a cell division, measured from n=6 different limbs. Median and quartiles are represented as dashed and dotted lines, respectively.

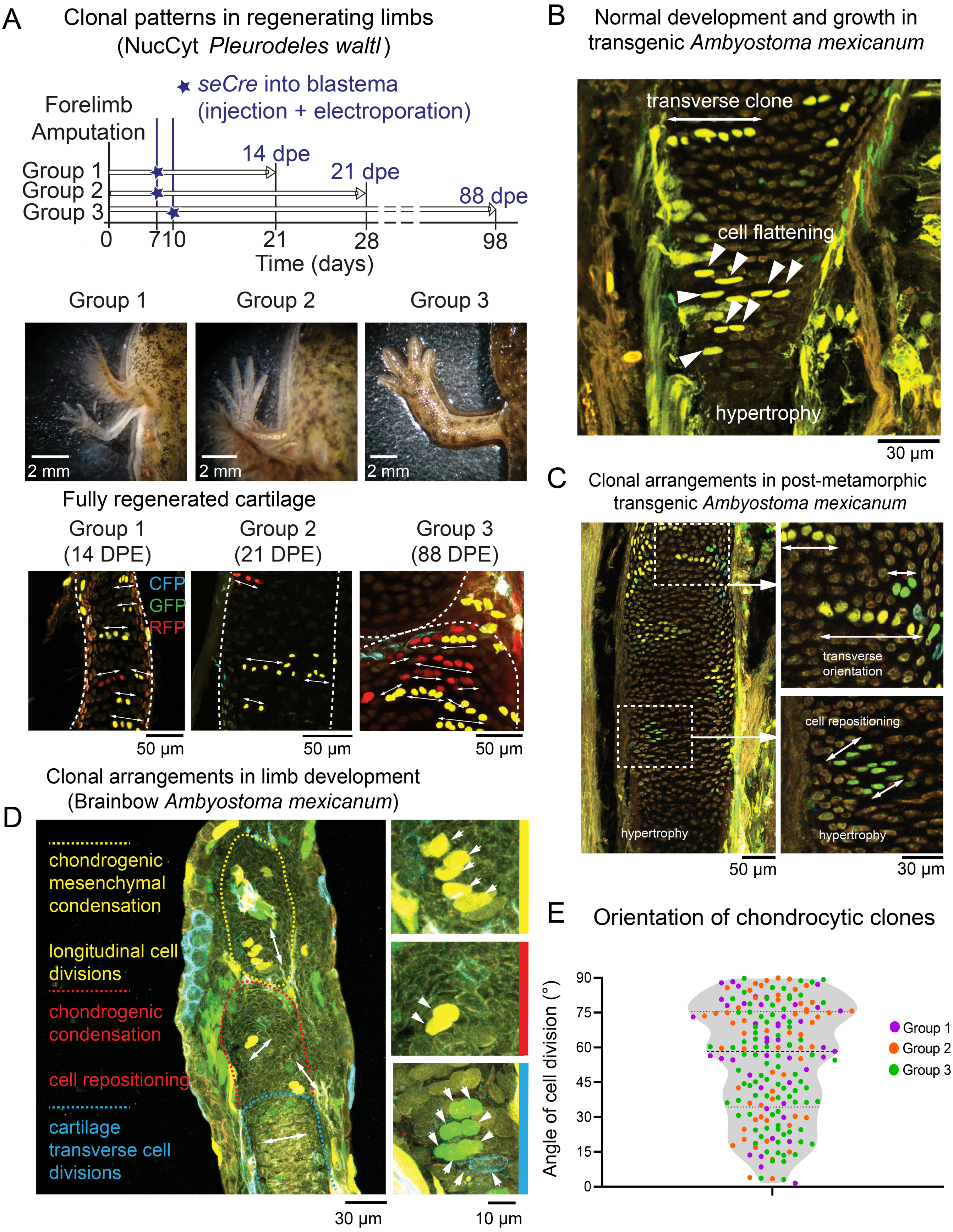
A
Clonal patterns in regenerating limbs (NucCyt Pleurodeles waltl)
seCre into blastema (injection + electroporation)
Forelimb Amputation
Group 1
Group 2
Group 3
14 dpe
21 dpe
88 dpe
0
7
10
21
28
98
Time (days)
Group 1
Group 2
Group 3
2 mm
Fully regenerated cartilage
Group 1 (14 DPE)
Group 2 (21 DPE)
Group 3 (88 DPE)
CFP
GFP
RFP
50 µm
Clonal arrangements in limb development (Brainbow Ambyostoma mexicanum)
D
chondrogenic mesenchymal condensation
longitudinal cell divisions
chondrogenic condensation
cell repositioning
cartilage transverse cell divisions
30 µm
10 µm
B
Normal development and growth in transgenic Ambyostoma mexicanum
transverse clone
cell flattening
hypertrophy
30 µm
C
Clonal arrangements in post-metamorphic transgenic Ambyostoma mexicanum
transverse orientation
cell repositioning
hypertrophy
50 µm
30 µm
E
Orientation of chondrocytic clones
Angle of cell division (°)
0
15
30
45
60
75
90
Group 1
Group 2
Group 3

Figure S6. Transversal orientation of chondrogenic clones occurs during the development and regeneration of rod-shaped cartilages in salamanders

(A) Experimental design for the study of clonal chondrocyte arrangements using genetic tracing in Nucbow-Cytbow (NucCyt) *Pleurodeles waltl*. Self-excising Cre (seCre) was injected into the blastema, and electroporation was performed. The limbs were collected after achieving complete regeneration at 14 (Group 1), 21 (Group 2) and 88 (Group 3) days post-electroporation (d.p.e.). Group 1 consisted of stage 54 larvae, group 2 were stage 55a larvae, and group 3 were post-metamorphic juveniles. Analysis of cell divisions patterns in the regenerating limbs shows prevalence of transverse cell division. We provide representative images of the animals used for this experiment and rod-shaped cartilages.

(B) Transverse orientation of chondrocytic clones in normally developing limb cartilages of *Ambystoma mexicanum* and flattened cells in pre-hypertrophic zone.

(C) Transversal clonal arrangements in post-metamorphic Brainbow *Ambystoma mexicanum* limb. Note the beginning of the onset of chondrocyte cell flattening, repositioning and hypertrophy.

(D) Genetic tracing in normally developing finger of Brainbow *Ambystoma mexicanum* larvae shows transverse cell divisions resulting in spatially arranged clones. Note changes in the orientation of traced clones: from longitudinal orientation within the mesenchymal condensation (color-coded as yellow) towards the transverse orientation in differentiating cartilage (blue dotted line). Yellow dotted line outlines the area of mesenchymal condensation, red dotted line marks the area of early chondrogenic differentiation, and the blue dotted line outlines the area of differentiated cartilage. Insets on the right side are either magnification of marked clones or a representative picture from a neighboring section of a corresponding area.

(E) Violin plot shows the quantification of cell division orientations in clones after complete regeneration in *Pleurodeles waltl* groups 1, 2 and 3. One-way ANOVA indicated no significant differences between groups (p=0.0958); therefore, all groups are plotted in a single graph. Note the predominant transverse orientation. Each data point represents the orientation of a cell division, measured from each of the 3 experimental groups. Group 1 (n=8 limbs); Group 2 (n=10 limbs); Group 3 (n=12 limbs). Median and quartiles are represented as dashed and dotted lines, respectively.

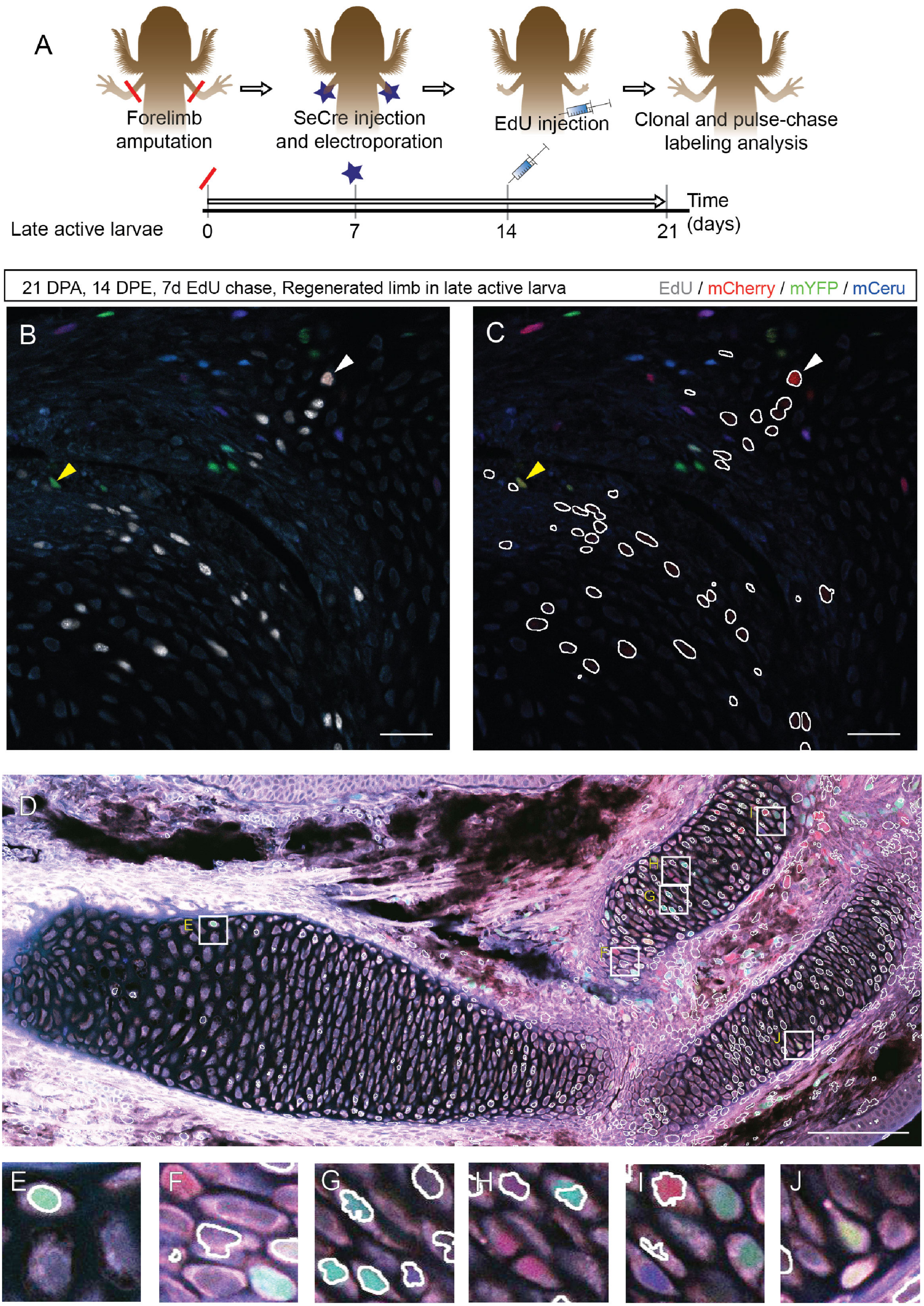
A
Forelimb amputation
SeCre injection and electroporation
EdU injection
Clonal and pulse-chase labeling analysis
Late active larvae
0
7
14
21
Time (days)
21 DPA, 14 DPE, 7d EdU chase, Regenerated limb in late active larva
EdU / mCherry / mYFP / mCeru
B
C
D
E
F
G
H
I
J

Figure S7. Combination of cell lineage tracing and Edu pulse-chase in *Pleurodeles* supports the lack of clonal expansion and the cell flattening/repositioning of cells

(A) Experimental outline of the experiment, where blastema cells were labelled by cre-activated clonal tracing followed by EdU pulse-chase.

(B) Representative image showing the combination of fluorescent proteins and EdU staining used for further processing.

(C) Example of the automated detection and visualisation image processing method used to analyse the EdU positive cells without compromising the colour-based analysis of NucCyt clones.

(D) Representative section of a regenerating limb showing the humerus, radius and ulna containing multiple labelled clones and 7 days-EdU chase.

(E-J) Higher magnification of the insets is depicted in (D). Note the absence of massive clonal expansion and the chondrocyte flattening followed by cell repositioning.

Scale bars: 50 µm (B,C); 200µm (D)

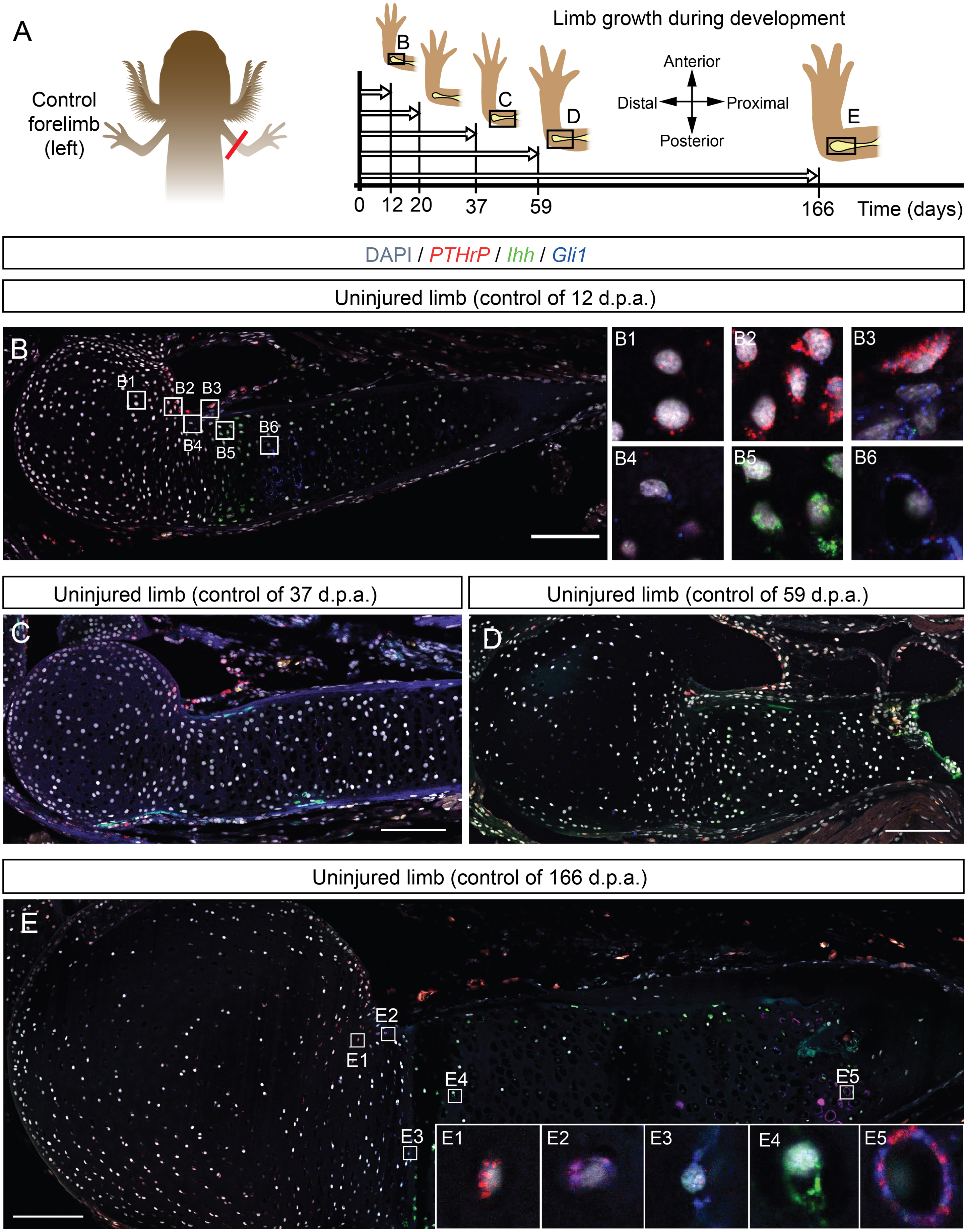
A
Control
forelimb
(left)
Limb growth during development
B
C
D
E
Anterior
Distal
Proximal
Posterior
0
12
20
37
59
166
Time (days)
DAPI / *PTHrP* / *Ihh* / *Gli1*
Uninjured limb (control of 12 d.p.a.)
B
B1
B2
B3
B4
B5
B6
Uninjured limb (control of 37 d.p.a.)
Uninjured limb (control of 59 d.p.a.)
C
D
Uninjured limb (control of 166 d.p.a.)
E
E1
E2
E3
E4
E5

Figure S8. The PTHrP-Ihh loop is expressed longitudinally in the developing humeral cartilage

(A) Experimental outline for the assessment of the *PTHrP-Ihh* loop during physiological growth in *Pleurodeles waltl*. Larvae at stage 54 received unilateral amputations. At the selected time points, the contralateral control limbs were collected for analysis (see Fig. 6 for the corresponding regenerating samples). The limbs used as controls and the region shown in B-E are drawn on top of the timeline. The axis applies to all images shown below (B-E).

(B) At 12 days post-amputation (d.p.a.), the contralateral control humerus´ articulation has acquired the rounded shape characteristic of the long bones. *PTHrP* expression was detected in round chondrocytes (B1, B2) as well as periskeletal cells (B3). Towards the proximal part of the developing bone, Gli1 positive puncta were detected (B3, B4), followed by an *Ihh* positive expression area (B5). The hypertrophic chondrocytes showed both *PTHrP* and *Gli1* puncta (B6). Scale bar, 200µm.

(C) At 37 d.p.a., the animals were collected around metamorphosis time. The main change in the contralateral control humerus compared to the previous time point (panel B) was a decrease in the Ihh positive expression area. *PTHrP* and *Gli1* expression was maintained. Scale bar, 200µm.

(D) At 59 d.p.a., similar patterns were detected compared to the previous timepoint (C) for *PTHrP, Gli1* and *Ihh* expression in the contralateral control humerus. Scale bar, 200µm.

(E) At 166 d.p.a., the contralateral control humerus had grown mainly through extracellular matrix production, as can be extrapolated by an increase in the distance between nuclei compared to previous time points. The *PTHrP, Gli1* and *Ihh* expression patterns were maintained (E1-E5). Scale bar, 200µm.
