# Supplementary material for: Altered developmental programs and oriented cell divisions lead to bulky bones during salamander limb regeneration
Source: Nat Commun. 2022 Nov 14;13:6949. doi: 10.1038/s41467-022-34266-w (PMC9663504; doi:10.1038/s41467-022-34266-w)
Supplement: Supplementary file 3 — Description of additional Supplementary File [file 41467_2022_34266_MOESM3_ESM.pdf]

Descriptions of Additional Supplementary Files

Supplementary data file 1
RNA in situ hybridisation experiments were performed using RNAscope® technology, and in this file, the complete information including sequences on the design of paired double-Z oligonucleotide probes against target RNA using custom software is provided.

Supplementary data file 2
This table lists all information on the animals used in this study: species, strain, stage, size, number of animals used per experiment, type of experimental procedure and analysis, *etc*.
